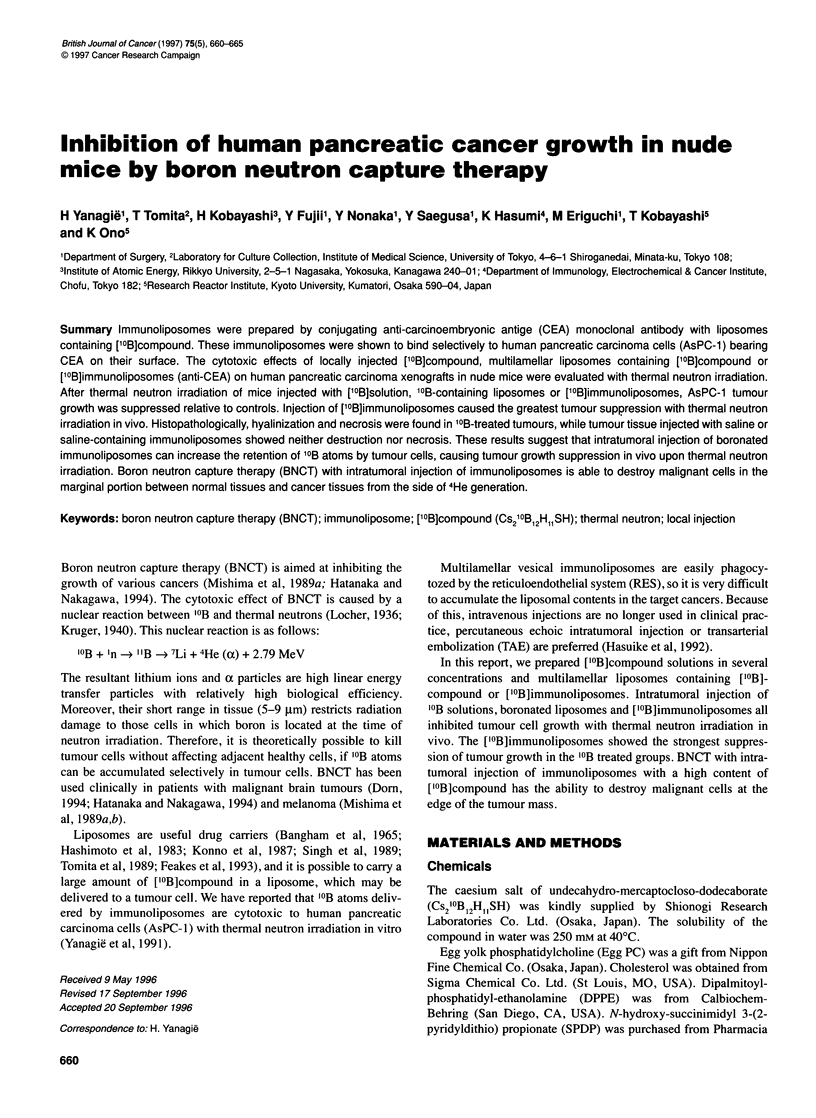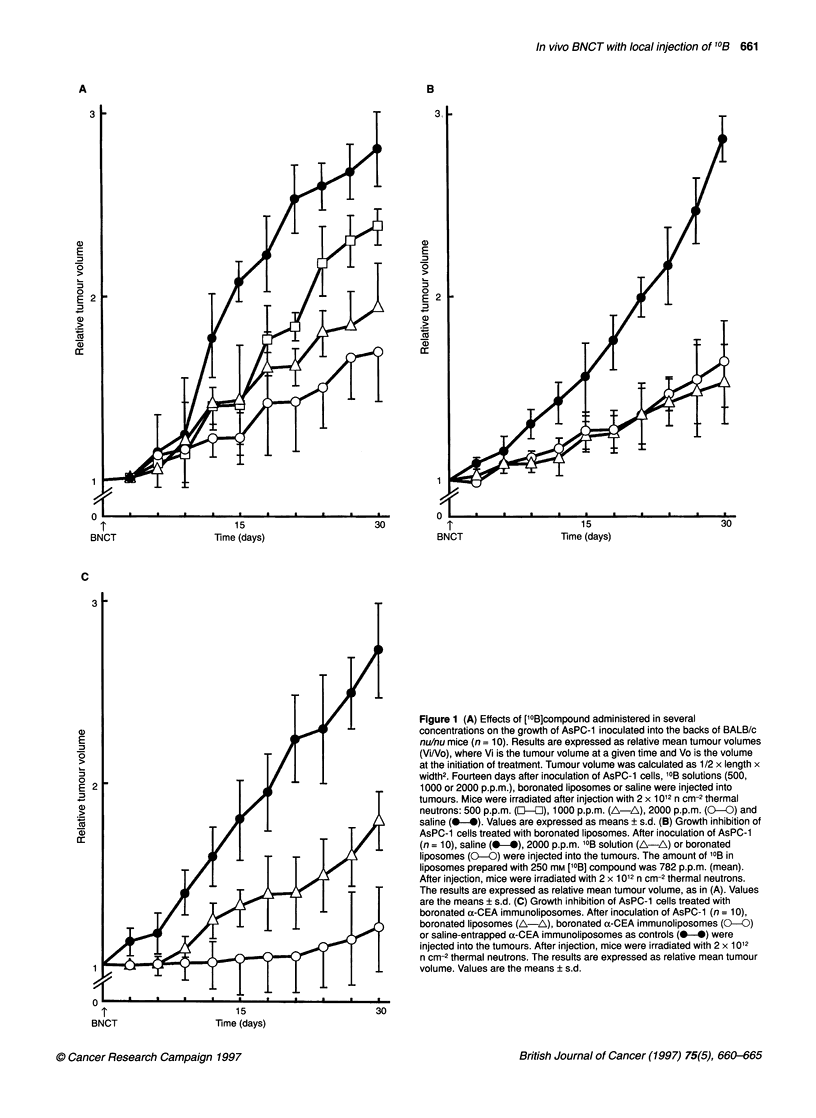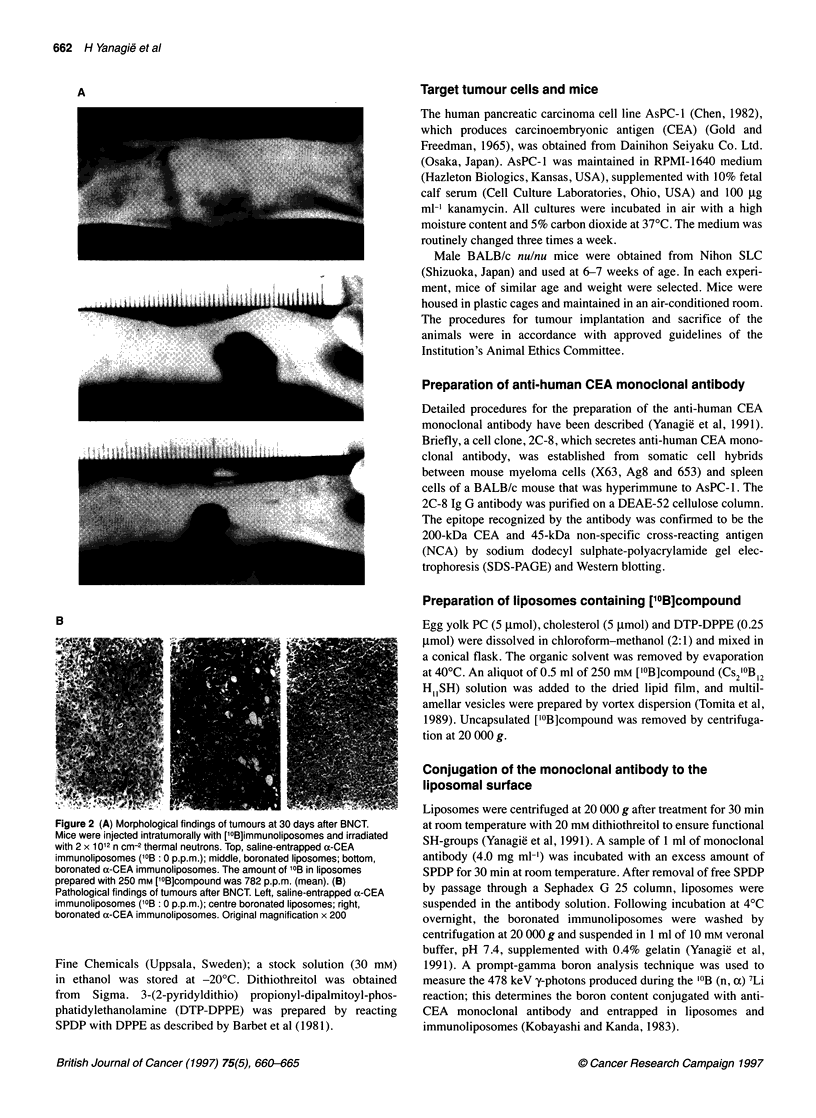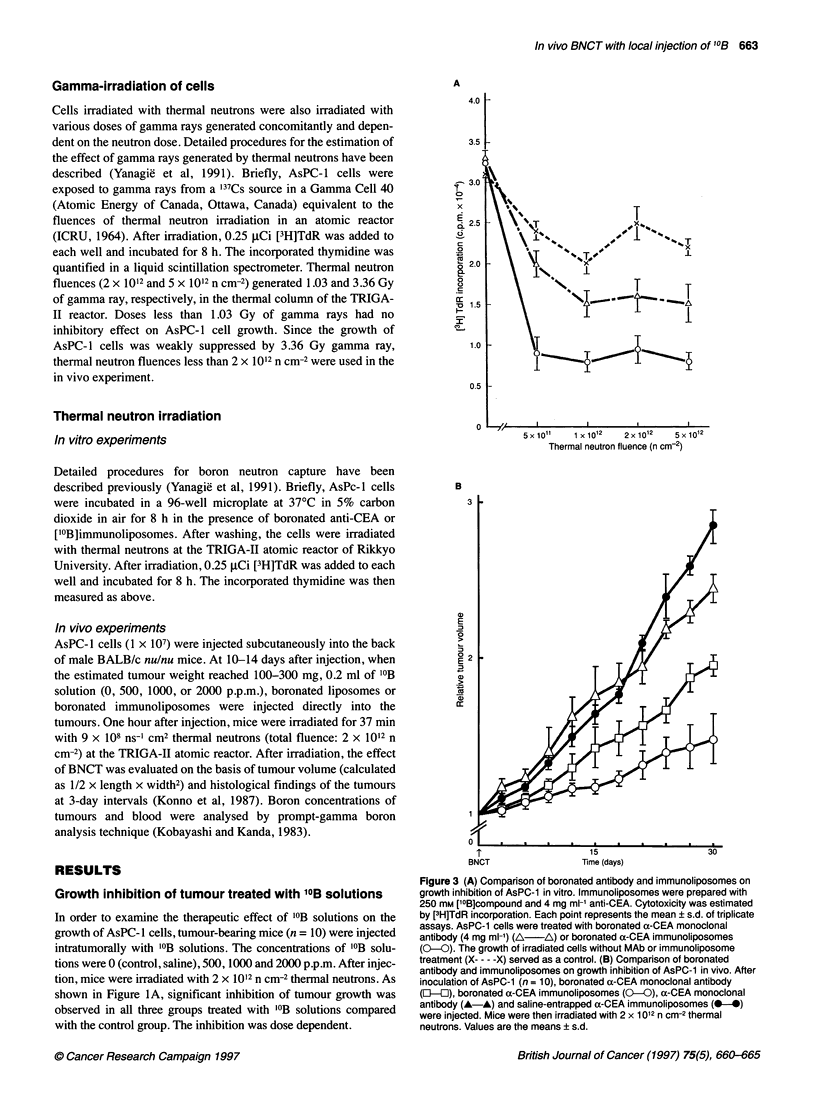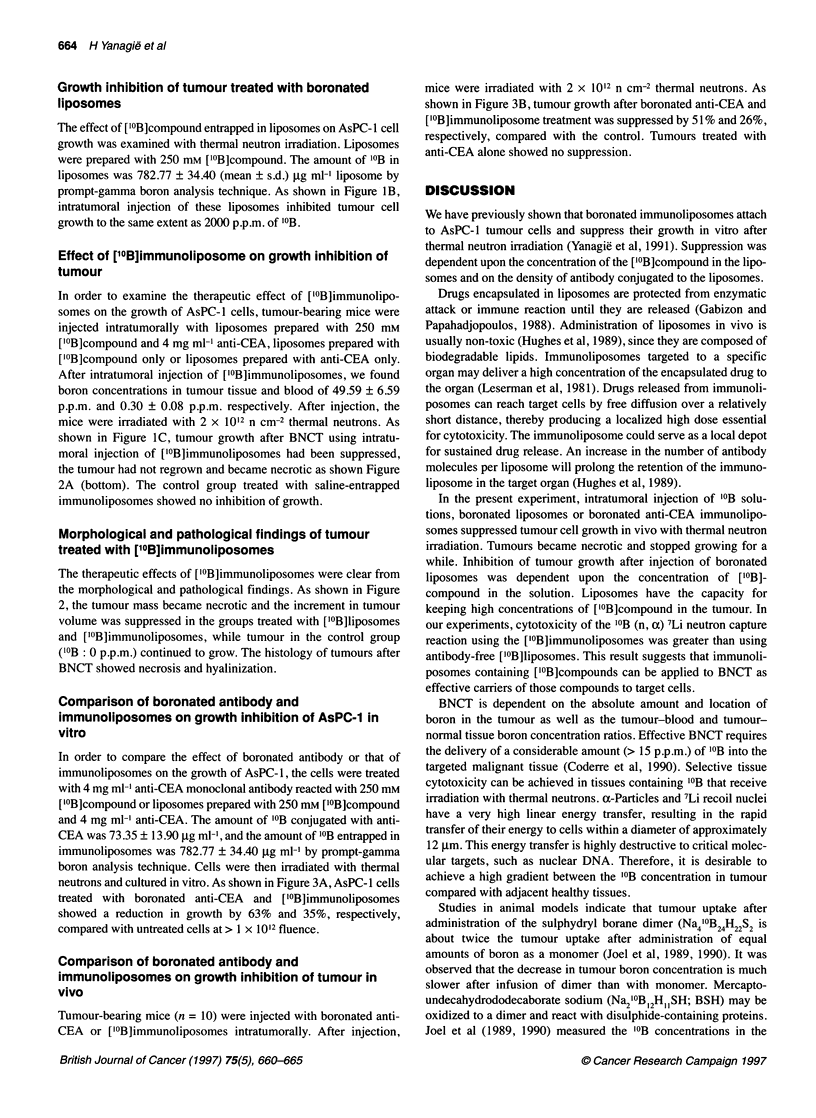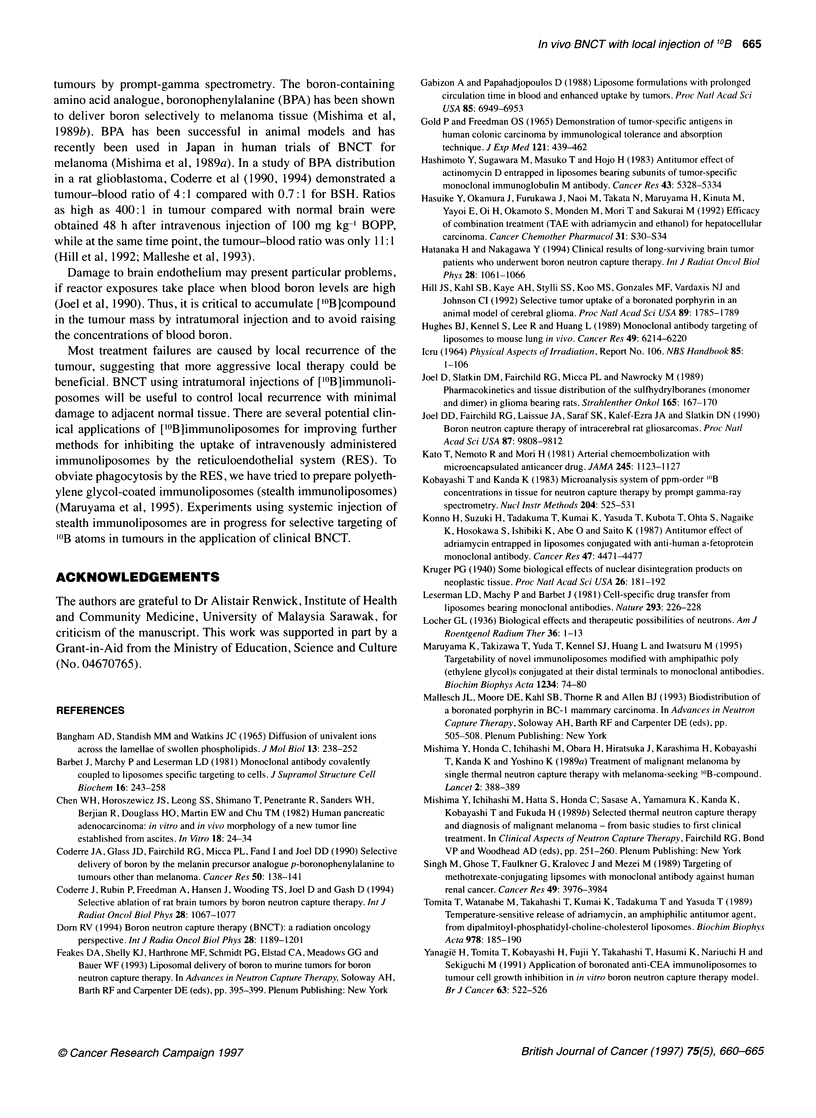# Inhibition of human pancreatic cancer growth in nude mice by boron neutron capture therapy.

**DOI:** 10.1038/bjc.1997.118

**Published:** 1997

**Authors:** H. Yanagië, T. Tomita, H. Kobayashi, Y. Fujii, Y. Nonaka, Y. Saegusa, K. Hasumi, M. Eriguchi, T. Kobayashi, K. Ono

**Affiliations:** Department of Surgery, Institute of Medical Science, University of Tokyo, Minata-ku, Japan.

## Abstract

**Images:**


British Journal of Cancer (1997) 75(5), 660–665
© 1997 Cancer Research Campaign

### Inhibition of human pancreatic cancer growth in nude mice by boron neutron capture therapy

**H Yanagië[1], T Tomita[2], H Kobayashi[3], Y Fujii[1], Y Nonaka[1], Y Saegusa[1], K Hasumi[4], M Eriguchi[1], T Kobayashi[5] and K Ono[5]**

[1]Department of Surgery, [2]Laboratory for Culture Collection, Institute of Medical Science, University of Tokyo, 4–6–1 Shiroganedai, Minata-ku, Tokyo 108; [3]Institute of Atomic Energy, Rikkyo University, 2–5–1 Nagasaka, Yokosuka, Kanagawa 240–01; [4]Department of Immunology, Electrochemical & Cancer Institute, Chofu, Tokyo 182; [5]Research Reactor Institute, Kyoto University, Kumatori, Osaka 590–04, Japan

**Summary** Immunoliposomes were prepared by conjugating anti-carcinoembryonic antige (CEA) monoclonal antibody with liposomes containing [$^{10}$B]compound. These immunoliposomes were shown to bind selectively to human pancreatic carcinoma cells (AsPC-1) bearing CEA on their surface. The cytotoxic effects of locally injected [$^{10}$B]compound, multilamellar liposomes containing [$^{10}$B]compound or [$^{10}$B]immunoliposomes (anti-CEA) on human pancreatic carcinoma xenografts in nude mice were evaluated with thermal neutron irradiation. After thermal neutron irradiation of mice injected with [$^{10}$B]solution, $^{10}$B-containing liposomes or [$^{10}$B]immunoliposomes, AsPC-1 tumour growth was suppressed relative to controls. Injection of [$^{10}$B]immunoliposomes caused the greatest tumour suppression with thermal neutron irradiation in vivo. Histopathologically, hyalinization and necrosis were found in $^{10}$B-treated tumours, while tumour tissue injected with saline or saline-containing immunoliposomes showed neither destruction nor necrosis. These results suggest that intratumoral injection of boronated immunoliposomes can increase the retention of $^{10}$B atoms by tumour cells, causing tumour growth suppression in vivo upon thermal neutron irradiation. Boron neutron capture therapy (BNCT) with intratumoral injection of immunoliposomes is able to destroy malignant cells in the marginal portion between normal tissues and cancer tissues from the side of $^4$He generation.

**Keywords:** boron neutron capture therapy (BNCT); immunoliposome; [$^{10}$B]compound ($Cs_2{}^{10}B_{12}H_{11}SH$); thermal neutron; local injection

Boron neutron capture therapy (BNCT) is aimed at inhibiting the growth of various cancers (Mishima et al, 1989*a;* Hatanaka and Nakagawa, 1994). The cytotoxic effect of BNCT is caused by a nuclear reaction between $^{10}$B and thermal neutrons (Locher, 1936; Kruger, 1940). This nuclear reaction is as follows:

$$^{10}B + {}^{1}n \rightarrow {}^{11}B \rightarrow {}^{7}Li + {}^{4}He\ (\alpha) + 2.79\ MeV$$

The resultant lithium ions and α particles are high linear energy transfer particles with relatively high biological efficiency. Moreover, their short range in tissue (5–9 μm) restricts radiation damage to those cells in which boron is located at the time of neutron irradiation. Therefore, it is theoretically possible to kill tumour cells without affecting adjacent healthy cells, if $^{10}$B atoms can be accumulated selectively in tumour cells. BNCT has been used clinically in patients with malignant brain tumours (Dorn, 1994; Hatanaka and Nakagawa, 1994) and melanoma (Mishima et al, 1989*a,b*).

Liposomes are useful drug carriers (Bangham et al, 1965; Hashimoto et al, 1983; Konno et al, 1987; Singh et al, 1989; Tomita et al, 1989; Feakes et al, 1993), and it is possible to carry a large amount of [$^{10}$B]compound in a liposome, which may be delivered to a tumour cell. We have reported that $^{10}$B atoms delivered by immunoliposomes are cytotoxic to human pancreatic carcinoma cells (AsPC-1) with thermal neutron irradiation in vitro (Yanagië et al, 1991).

*Received 9 May 1996*
*Revised 17 September 1996*
*Accepted 20 September 1996*

*Correspondence to:* H. Yanagië

Multilamellar vesical immunoliposomes are easily phagocytozed by the reticuloendothelial system (RES), so it is very difficult to accumulate the liposomal contents in the target cancers. Because of this, intravenous injections are no longer used in clinical practice, percutaneous echoic intratumoral injection or transarterial embolization (TAE) are preferred (Hasuike et al, 1992).

In this report, we prepared [$^{10}$B]compound solutions in several concentrations and multilamellar liposomes containing [$^{10}$B]-compound or [$^{10}$B]immunoliposomes. Intratumoral injection of $^{10}$B solutions, boronated liposomes and [$^{10}$B]immunoliposomes all inhibited tumour cell growth with thermal neutron irradiation in vivo. The [$^{10}$B]immunoliposomes showed the strongest suppression of tumour growth in the $^{10}$B treated groups. BNCT with intratumoral injection of immunoliposomes with a high content of [$^{10}$B]compound has the ability to destroy malignant cells at the edge of the tumour mass.

### MATERIALS AND METHODS

### Chemicals

The caesium salt of undecahydro-mercaptocloso-dodecaborate ($Cs_2{}^{10}B_{12}H_{11}SH$) was kindly supplied by Shionogi Research Laboratories Co. Ltd. (Osaka, Japan). The solubility of the compound in water was 250 mM at 40°C.

Egg yolk phosphatidylcholine (Egg PC) was a gift from Nippon Fine Chemical Co. (Osaka, Japan). Cholesterol was obtained from Sigma Chemical Co. Ltd. (St Louis, MO, USA). Dipalmitoyl-phosphatidyl-ethanolamine (DPPE) was from Calbiochem-Behring (San Diego, CA, USA). *N*-hydroxy-succinimidyl 3-(2-pyridyldithio) propionate (SPDP) was purchased from Pharmacia

**Figure 1** (**A**) Effects of [$^{10}$B]compound administered in several concentrations on the growth of AsPC-1 inoculated into the backs of BALB/c *nu/nu* mice ($n$ = 10). Results are expressed as relative mean tumour volumes (Vi/Vo), where Vi is the tumour volume at a given time and Vo is the volume at the initiation of treatment. Tumour volume was calculated as 1/2 × length × width$^2$. Fourteen days after inoculation of AsPC-1 cells, $^{10}$B solutions (500, 1000 or 2000 p.p.m.), boronated liposomes or saline were injected into tumours. Mice were irradiated after injection with 2 × 10$^{12}$ n cm$^{-2}$ thermal neutrons: 500 p.p.m. (□—□), 1000 p.p.m. (△—△), 2000 p.p.m. (○—○) and saline (●—●). Values are expressed as means ± s.d. (**B**) Growth inhibition of AsPC-1 cells treated with boronated liposomes. After inoculation of AsPC-1 ($n$ = 10), saline (●—●), 2000 p.p.m. $^{10}$B solution (△—△) or boronated liposomes (○—○) were injected into the tumours. The amount of $^{10}$B in liposomes prepared with 250 mM [$^{10}$B] compound was 782 p.p.m. (mean). After injection, mice were irradiated with 2 × 10$^{12}$ n cm$^{-2}$ thermal neutrons. The results are expressed as relative mean tumour volume, as in (A). Values are the means ± s.d. (**C**) Growth inhibition of AsPC-1 cells treated with boronated α-CEA immunoliposomes. After inoculation of AsPC-1 ($n$ = 10), boronated liposomes (△—△), boronated α-CEA immunoliposomes (○—○) or saline-entrapped α-CEA immunoliposomes as controls (●—●) were injected into the tumours. After injection, mice were irradiated with 2 × 10$^{12}$ n cm$^{-2}$ thermal neutrons. The results are expressed as relative mean tumour volume. Values are the means ± s.d.

Figure 2 (A) Morphological findings of tumours at 30 days after BNCT. Mice were injected intratumorally with [$^{10}$B]immunoliposomes and irradiated with 2 × $10^{12}$ n $cm^{-2}$ thermal neutrons. Top, saline-entrapped α-CEA immunoliposomes ($^{10}$B : 0 p.p.m.); middle, boronated liposomes; bottom, boronated α-CEA immunoliposomes. The amount of $^{10}$B in liposomes prepared with 250 mM [$^{10}$B]compound was 782 p.p.m. (mean). (B) Pathological findings of tumours after BNCT. Left, saline-entrapped α-CEA immunoliposomes ($^{10}$B : 0 p.p.m.); centre boronated liposomes; right, boronated α-CEA immunoliposomes. Original magnification × 200

Fine Chemicals (Uppsala, Sweden); a stock solution (30 mM) in ethanol was stored at –20°C. Dithiothreitol was obtained from Sigma. 3-(2-pyridyldithio) propionyl-dipalmitoyl-phosphatidylethanolamine (DTP-DPPE) was prepared by reacting SPDP with DPPE as described by Barbet et al (1981).

### Target tumour cells and mice

The human pancreatic carcinoma cell line AsPC-1 (Chen, 1982), which produces carcinoembryonic antigen (CEA) (Gold and Freedman, 1965), was obtained from Dainihon Seiyaku Co. Ltd. (Osaka, Japan). AsPC-1 was maintained in RPMI-1640 medium (Hazleton Biologics, Kansas, USA), supplemented with 10% fetal calf serum (Cell Culture Laboratories, Ohio, USA) and 100 μg $ml^{-1}$ kanamycin. All cultures were incubated in air with a high moisture content and 5% carbon dioxide at 37°C. The medium was routinely changed three times a week.

Male BALB/c *nu/nu* mice were obtained from Nihon SLC (Shizuoka, Japan) and used at 6–7 weeks of age. In each experiment, mice of similar age and weight were selected. Mice were housed in plastic cages and maintained in an air-conditioned room. The procedures for tumour implantation and sacrifice of the animals were in accordance with approved guidelines of the Institution's Animal Ethics Committee.

### Preparation of anti-human CEA monoclonal antibody

Detailed procedures for the preparation of the anti-human CEA monoclonal antibody have been described (Yanagië et al, 1991). Briefly, a cell clone, 2C-8, which secretes anti-human CEA monoclonal antibody, was established from somatic cell hybrids between mouse myeloma cells (X63, Ag8 and 653) and spleen cells of a BALB/c mouse that was hyperimmune to AsPC-1. The 2C-8 Ig G antibody was purified on a DEAE-52 cellulose column. The epitope recognized by the antibody was confirmed to be the 200-kDa CEA and 45-kDa non-specific cross-reacting antigen (NCA) by sodium dodecyl sulphate-polyacrylamide gel electrophoresis (SDS-PAGE) and Western blotting.

### Preparation of liposomes containing [$^{10}$B]compound

Egg yolk PC (5 μmol), cholesterol (5 μmol) and DTP-DPPE (0.25 μmol) were dissolved in chloroform–methanol (2:1) and mixed in a conical flask. The organic solvent was removed by evaporation at 40°C. An aliquot of 0.5 ml of 250 mM [$^{10}$B]compound ($Cs_2{}^{10}B_{12}H_{11}SH$) solution was added to the dried lipid film, and multilamellar vesicles were prepared by vortex dispersion (Tomita et al, 1989). Uncapsulated [$^{10}$B]compound was removed by centrifugation at 20 000 ***g***.

### Conjugation of the monoclonal antibody to the liposomal surface

Liposomes were centrifuged at 20 000 ***g*** after treatment for 30 min at room temperature with 20 mM dithiothreitol to ensure functional SH-groups (Yanagië et al, 1991). A sample of 1 ml of monoclonal antibody (4.0 mg $ml^{-1}$) was incubated with an excess amount of SPDP for 30 min at room temperature. After removal of free SPDP by passage through a Sephadex G 25 column, liposomes were suspended in the antibody solution. Following incubation at 4°C overnight, the boronated immunoliposomes were washed by centrifugation at 20 000 ***g*** and suspended in 1 ml of 10 mM veronal buffer, pH 7.4, supplemented with 0.4% gelatin (Yanagië et al, 1991). A prompt-gamma boron analysis technique was used to measure the 478 keV γ-photons produced during the $^{10}$B (n, α) $^7$Li reaction; this determines the boron content conjugated with anti-CEA monoclonal antibody and entrapped in liposomes and immunoliposomes (Kobayashi and Kanda, 1983).

© Cancer Research Campaign 1997

### Gamma-irradiation of cells

Cells irradiated with thermal neutrons were also irradiated with various doses of gamma rays generated concomitantly and dependent on the neutron dose. Detailed procedures for the estimation of the effect of gamma rays generated by thermal neutrons have been described (Yanagië et al, 1991). Briefly, AsPC-1 cells were exposed to gamma rays from a $^{137}Cs$ source in a Gamma Cell 40 (Atomic Energy of Canada, Ottawa, Canada) equivalent to the fluences of thermal neutron irradiation in an atomic reactor (ICRU, 1964). After irradiation, 0.25 μCi [$^{3}H$]TdR was added to each well and incubated for 8 h. The incorporated thymidine was quantified in a liquid scintillation spectrometer. Thermal neutron fluences ($2 \times 10^{12}$ and $5 \times 10^{12}$ n $cm^{-2}$) generated 1.03 and 3.36 Gy of gamma ray, respectively, in the thermal column of the TRIGA-II reactor. Doses less than 1.03 Gy of gamma rays had no inhibitory effect on AsPC-1 cell growth. Since the growth of AsPC-1 cells was weakly suppressed by 3.36 Gy gamma ray, thermal neutron fluences less than $2 \times 10^{12}$ n $cm^{-2}$ were used in the in vivo experiment.

### Thermal neutron irradiation

#### *In vitro experiments*

Detailed procedures for boron neutron capture have been described previously (Yanagië et al, 1991). Briefly, AsPc-1 cells were incubated in a 96-well microplate at 37°C in 5% carbon dioxide in air for 8 h in the presence of boronated anti-CEA or [$^{10}B$]immunoliposomes. After washing, the cells were irradiated with thermal neutrons at the TRIGA-II atomic reactor of Rikkyo University. After irradiation, 0.25 μCi [$^{3}H$]TdR was added to each well and incubated for 8 h. The incorporated thymidine was then measured as above.

#### *In vivo experiments*

AsPC-1 cells ($1 \times 10^{7}$) were injected subcutaneously into the back of male BALB/c *nu/nu* mice. At 10–14 days after injection, when the estimated tumour weight reached 100–300 mg, 0.2 ml of $^{10}B$ solution (0, 500, 1000, or 2000 p.p.m.), boronated liposomes or boronated immunoliposomes were injected directly into the tumours. One hour after injection, mice were irradiated for 37 min with $9 \times 10^{8}$ $ns^{-1}$ $cm^{2}$ thermal neutrons (total fluence: $2 \times 10^{12}$ n $cm^{-2}$) at the TRIGA-II atomic reactor. After irradiation, the effect of BNCT was evaluated on the basis of tumour volume (calculated as 1/2 × length × width$^{2}$) and histological findings of the tumours at 3-day intervals (Konno et al, 1987). Boron concentrations of tumours and blood were analysed by prompt-gamma boron analysis technique (Kobayashi and Kanda, 1983).

### RESULTS

### Growth inhibition of tumour treated with $^{10}B$ solutions

In order to examine the therapeutic effect of $^{10}B$ solutions on the growth of AsPC-1 cells, tumour-bearing mice ($n = 10$) were injected intratumorally with $^{10}B$ solutions. The concentrations of $^{10}B$ solutions were 0 (control, saline), 500, 1000 and 2000 p.p.m. After injection, mice were irradiated with $2 \times 10^{12}$ n $cm^{-2}$ thermal neutrons. As shown in Figure 1A, significant inhibition of tumour growth was observed in all three groups treated with $^{10}B$ solutions compared with the control group. The inhibition was dose dependent.

**Figure 3** (**A**) Comparison of boronated antibody and immunoliposomes on growth inhibition of AsPC-1 in vitro. Immunoliposomes were prepared with 250 mM [$^{10}B$]compound and 4 mg $ml^{-1}$ anti-CEA. Cytotoxicity was estimated by [$^{3}H$]TdR incorporation. Each point represents the mean ± s.d. of triplicate assays. AsPC-1 cells were treated with boronated α-CEA monoclonal antibody (4 mg $ml^{-1}$) (△——△) or boronated α-CEA immunoliposomes (○—○). The growth of irradiated cells without MAb or immunoliposome treatment (X- - - -X) served as a control. (**B**) Comparison of boronated antibody and immunoliposomes on growth inhibition of AsPC-1 in vivo. After inoculation of AsPC-1 ($n = 10$), boronated α-CEA monoclonal antibody (□—□), boronated α-CEA immunoliposomes (○—○), α-CEA monoclonal antibody (▲—▲) and saline-entrapped α-CEA immunoliposomes (●—●) were injected. Mice were then irradiated with $2 \times 10^{12}$ n $cm^{-2}$ thermal neutrons. Values are the means ± s.d.

© Cancer Research Campaign 1997

### Growth inhibition of tumour treated with boronated liposomes

The effect of [$^{10}$B]compound entrapped in liposomes on AsPC-1 cell growth was examined with thermal neutron irradiation. Liposomes were prepared with 250 mM [$^{10}$B]compound. The amount of $^{10}$B in liposomes was 782.77 ± 34.40 (mean ± s.d.) μg ml$^{-1}$ liposome by prompt-gamma boron analysis technique. As shown in Figure 1B, intratumoral injection of these liposomes inhibited tumour cell growth to the same extent as 2000 p.p.m. of $^{10}$B.

### Effect of [$^{10}$B]immunoliposome on growth inhibition of tumour

In order to examine the therapeutic effect of [$^{10}$B]immunoliposomes on the growth of AsPC-1 cells, tumour-bearing mice were injected intratumorally with liposomes prepared with 250 mM [$^{10}$B]compound and 4 mg ml$^{-1}$ anti-CEA, liposomes prepared with [$^{10}$B]compound only or liposomes prepared with anti-CEA only. After intratumoral injection of [$^{10}$B]immunoliposomes, we found boron concentrations in tumour tissue and blood of 49.59 ± 6.59 p.p.m. and 0.30 ± 0.08 p.p.m. respectively. After injection, the mice were irradiated with 2 × 10$^{12}$ n cm$^{-2}$ thermal neutrons. As shown in Figure 1C, tumour growth after BNCT using intratumoral injection of [$^{10}$B]immunoliposomes had been suppressed, the tumour had not regrown and became necrotic as shown Figure 2A (bottom). The control group treated with saline-entrapped immunoliposomes showed no inhibition of growth.

### Morphological and pathological findings of tumour treated with [$^{10}$B]immunoliposomes

The therapeutic effects of [$^{10}$B]immunoliposomes were clear from the morphological and pathological findings. As shown in Figure 2, the tumour mass became necrotic and the increment in tumour volume was suppressed in the groups treated with [$^{10}$B]liposomes and [$^{10}$B]immunoliposomes, while tumour in the control group ($^{10}$B : 0 p.p.m.) continued to grow. The histology of tumours after BNCT showed necrosis and hyalinization.

### Comparison of boronated antibody and immunoliposomes on growth inhibition of AsPC-1 in vitro

In order to compare the effect of boronated antibody or that of immunoliposomes on the growth of AsPC-1, the cells were treated with 4 mg ml$^{-1}$ anti-CEA monoclonal antibody reacted with 250 mM [$^{10}$B]compound or liposomes prepared with 250 mM [$^{10}$B]compound and 4 mg ml$^{-1}$ anti-CEA. The amount of $^{10}$B conjugated with anti-CEA was 73.35 ± 13.90 μg ml$^{-1}$, and the amount of $^{10}$B entrapped in immunoliposomes was 782.77 ± 34.40 μg ml$^{-1}$ by prompt-gamma boron analysis technique. Cells were then irradiated with thermal neutrons and cultured in vitro. As shown in Figure 3A, AsPC-1 cells treated with boronated anti-CEA and [$^{10}$B]immunoliposomes showed a reduction in growth by 63% and 35%, respectively, compared with untreated cells at > 1 × 10$^{12}$ fluence.

### Comparison of boronated antibody and immunoliposomes on growth inhibition of tumour in vivo

Tumour-bearing mice ($n$ = 10) were injected with boronated anti-CEA or [$^{10}$B]immunoliposomes intratumorally. After injection, mice were irradiated with 2 × 10$^{12}$ n cm$^{-2}$ thermal neutrons. As shown in Figure 3B, tumour growth after boronated anti-CEA and [$^{10}$B]immunoliposome treatment was suppressed by 51% and 26%, respectively, compared with the control. Tumours treated with anti-CEA alone showed no suppression.

### DISCUSSION

We have previously shown that boronated immunoliposomes attach to AsPC-1 tumour cells and suppress their growth in vitro after thermal neutron irradiation (Yanagië et al, 1991). Suppression was dependent upon the concentration of the [$^{10}$B]compound in the liposomes and on the density of antibody conjugated to the liposomes.

Drugs encapsulated in liposomes are protected from enzymatic attack or immune reaction until they are released (Gabizon and Papahadjopoulos, 1988). Administration of liposomes in vivo is usually non-toxic (Hughes et al, 1989), since they are composed of biodegradable lipids. Immunoliposomes targeted to a specific organ may deliver a high concentration of the encapsulated drug to the organ (Leserman et al, 1981). Drugs released from immunoliposomes can reach target cells by free diffusion over a relatively short distance, thereby producing a localized high dose essential for cytotoxicity. The immunoliposome could serve as a local depot for sustained drug release. An increase in the number of antibody molecules per liposome will prolong the retention of the immunoliposome in the target organ (Hughes et al, 1989).

In the present experiment, intratumoral injection of $^{10}$B solutions, boronated liposomes or boronated anti-CEA immunoliposomes suppressed tumour cell growth in vivo with thermal neutron irradiation. Tumours became necrotic and stopped growing for a while. Inhibition of tumour growth after injection of boronated liposomes was dependent upon the concentration of [$^{10}$B]-compound in the solution. Liposomes have the capacity for keeping high concentrations of [$^{10}$B]compound in the tumour. In our experiments, cytotoxicity of the $^{10}$B (n, α) $^{7}$Li neutron capture reaction using the [$^{10}$B]immunoliposomes was greater than using antibody-free [$^{10}$B]liposomes. This result suggests that immunoliposomes containing [$^{10}$B]compounds can be applied to BNCT as effective carriers of those compounds to target cells.

BNCT is dependent on the absolute amount and location of boron in the tumour as well as the tumour–blood and tumour–normal tissue boron concentration ratios. Effective BNCT requires the delivery of a considerable amount (> 15 p.p.m.) of $^{10}$B into the targeted malignant tissue (Coderre et al, 1990). Selective tissue cytotoxicity can be achieved in tissues containing $^{10}$B that receive irradiation with thermal neutrons. α-Particles and $^{7}$Li recoil nuclei have a very high linear energy transfer, resulting in the rapid transfer of their energy to cells within a diameter of approximately 12 μm. This energy transfer is highly destructive to critical molecular targets, such as nuclear DNA. Therefore, it is desirable to achieve a high gradient between the $^{10}$B concentration in tumour compared with adjacent healthy tissues.

Studies in animal models indicate that tumour uptake after administration of the sulphydryl borane dimer ($Na_4{}^{10}B_{24}H_{22}S_2$ is about twice the tumour uptake after administration of equal amounts of boron as a monomer (Joel et al, 1989, 1990). It was observed that the decrease in tumour boron concentration is much slower after infusion of dimer than with monomer. Mercapto-undecahydrododecaborate sodium ($Na_2{}^{10}B_{12}H_{11}SH$; BSH) may be oxidized to a dimer and react with disulphide-containing proteins. Joel et al (1989, 1990) measured the $^{10}$B concentrations in the

© Cancer Research Campaign 1997

tumours by prompt-gamma spectrometry. The boron-containing amino acid analogue, boronophenylalanine (BPA) has been shown to deliver boron selectively to melanoma tissue (Mishima et al, 1989*b*). BPA has been successful in animal models and has recently been used in Japan in human trials of BNCT for melanoma (Mishima et al, 1989*a*). In a study of BPA distribution in a rat glioblastoma, Coderre et al (1990, 1994) demonstrated a tumour–blood ratio of 4:1 compared with 0.7:1 for BSH. Ratios as high as 400:1 in tumour compared with normal brain were obtained 48 h after intravenous injection of 100 mg $kg^{-1}$ BOPP, while at the same time point, the tumour–blood ratio was only 11:1 (Hill et al, 1992; Malleshe et al, 1993).

Damage to brain endothelium may present particular problems, if reactor exposures take place when blood boron levels are high (Joel et al, 1990). Thus, it is critical to accumulate [$^{10}B$]compound in the tumour mass by intratumoral injection and to avoid raising the concentrations of blood boron.

Most treatment failures are caused by local recurrence of the tumour, suggesting that more aggressive local therapy could be beneficial. BNCT using intratumoral injections of [$^{10}B$]immunoliposomes will be useful to control local recurrence with minimal damage to adjacent normal tissue. There are several potential clinical applications of [$^{10}B$]immunoliposomes for improving further methods for inhibiting the uptake of intravenously administered immunoliposomes by the reticuloendothelial system (RES). To obviate phagocytosis by the RES, we have tried to prepare polyethylene glycol-coated immunoliposomes (stealth immunoliposomes) (Maruyama et al, 1995). Experiments using systemic injection of stealth immunoliposomes are in progress for selective targeting of $^{10}B$ atoms in tumours in the application of clinical BNCT.

### ACKNOWLEDGEMENTS

The authors are grateful to Dr Alistair Renwick, Institute of Health and Community Medicine, University of Malaysia Sarawak, for criticism of the manuscript. This work was supported in part by a Grant-in-Aid from the Ministry of Education, Science and Culture (No. 04670765).

### REFERENCES

Bangham AD, Standish MM and Watkins JC (1965) Diffusion of univalent ions across the lamellae of swollen phospholipids. *J Mol Biol* **13**: 238–252

Barbet J, Marchy P and Leserman LD (1981) Monoclonal antibody covalently coupled to liposomes specific targeting to cells. *J Supramol Structure Cell Biochem* **16**: 243–258

Chen WH, Horoszewicz JS, Leong SS, Shimano T, Penetrante R, Sanders WH, Berjian R, Douglass HO, Martin EW and Chu TM (1982) Human pancreatic adenocarcinoma: *in vitro* and *in vivo* morphology of a new tumor line established from ascites. *In Vitro* **18**: 24–34

Coderre JA, Glass JD, Fairchild RG, Micca PL, Fand I and Joel DD (1990) Selective delivery of boron by the melanin precursor analogue *p*-boronophenylalanine to tumours other than melanoma. *Cancer Res* **50**: 138–141

Coderre J, Rubin P, Freedman A, Hansen J, Wooding TS, Joel D and Gash D (1994) Selective ablation of rat brain tumors by boron neutron capture therapy. *Int J Radiat Oncol Biol Phys* **28**: 1067–1077

Dorn RV (1994) Boron neutron capture therapy (BNCT): a radiation oncology perspective. *Int J Radia Oncol Biol Phys* **28**: 1189–1201

Feakes DA, Shelly KJ, Harthrone MF, Schmidt PG, Elstad CA, Meadows GG and Bauer WF (1993) Liposomal delivery of boron to murine tumors for boron neutron capture therapy. In *Advances in Neutron Capture Therapy*, Soloway AH, Barth RF and Carpenter DE (eds), pp. 395–399. Plenum Publishing: New York

Gabizon A and Papahadjopoulos D (1988) Liposome formulations with prolonged circulation time in blood and enhanced uptake by tumors. *Proc Natl Acad Sci USA* **85**: 6949–6953

Gold P and Freedman OS (1965) Demonstration of tumor-specific antigens in human colonic carcinoma by immunological tolerance and absorption technique. *J Exp Med* **121**: 439–462

Hashimoto Y, Sugawara M, Masuko T and Hojo H (1983) Antitumor effect of actinomycin D entrapped in liposomes bearing subunits of tumor-specific monoclonal immunoglobulin M antibody. *Cancer Res* **43**: 5328–5334

Hasuike Y, Okamura J, Furukawa J, Naoi M, Takata N, Maruyama H, Kinuta M, Yayoi E, Oi H, Okamoto S, Monden M, Mori T and Sakurai M (1992) Efficacy of combination treatment (TAE with adriamycin and ethanol) for hepatocellular carcinoma. *Cancer Chemother Pharmacol* **31**: S30–S34

Hatanaka H and Nakagawa Y (1994) Clinical results of long-surviving brain tumor patients who underwent boron neutron capture therapy. *Int J Radiat Oncol Biol Phys* **28**: 1061–1066

Hill JS, Kahl SB, Kaye AH, Stylli SS, Koo MS, Gonzales MF, Vardaxis NJ and Johnson CI (1992) Selective tumor uptake of a boronated porphyrin in an animal model of cerebral glioma. *Proc Natl Acad Sci USA* **89**: 1785–1789

Hughes BJ, Kennel S, Lee R and Huang L (1989) Monoclonal antibody targeting of liposomes to mouse lung *in vivo*. *Cancer Res* **49**: 6214–6220

Icru (1964) *Physical Aspects of Irradiation*, Report No. 106. *NBS Handbook* **85**: 1-106

Joel D, Slatkin DM, Fairchild RG, Micca PL and Nawrocky M (1989) Pharmacokinetics and tissue distribution of the sulfhydrylboranes (monomer and dimer) in glioma bearing rats. *Strahlenther Onkol* **165**: 167–170

Joel DD, Fairchild RG, Laissue JA, Saraf SK, Kalef-Ezra JA and Slatkin DN (1990) Boron neutron capture therapy of intracerebral rat gliosarcomas. *Proc Natl Acad Sci USA* **87**: 9808–9812

Kato T, Nemoto R and Mori H (1981) Arterial chemoembolization with microencapsulated anticancer drug. *JAMA* **245**: 1123–1127

Kobayashi T and Kanda K (1983) Microanalysis system of ppm-order $^{10}B$ concentrations in tissue for neutron capture therapy by prompt gamma-ray spectrometry. *Nucl Instr Methods* **204**: 525–531

Konno H, Suzuki H, Tadakuma T, Kumai K, Yasuda T, Kubota T, Ohta S, Nagaike K, Hosokawa S, Ishibiki K, Abe O and Saito K (1987) Antitumor effect of adriamycin entrapped in liposomes conjugated with anti-human a-fetoprotein monoclonal antibody. *Cancer Res* **47**: 4471–4477

Kruger PG (1940) Some biological effects of nuclear disintegration products on neoplastic tissue. *Proc Natl Acad Sci USA* **26**: 181–192

Leserman LD, Machy P and Barbet J (1981) Cell-specific drug transfer from liposomes bearing monoclonal antibodies. *Nature* **293**: 226–228

Locher GL (1936) Biological effects and therapeutic possibilities of neutrons. *Am J Roentgenol Radium Ther* **36**: 1–13

Maruyama K, Takizawa T, Yuda T, Kennel SJ, Huang L and Iwatsuru M (1995) Targetability of novel immunoliposomes modified with amphipathic poly (ethylene glycol)s conjugated at their distal terminals to monoclonal antibodies. *Biochim Biophys Acta* **1234**: 74–80

Mallesch JL, Moore DE, Kahl SB, Thorne R and Allen BJ (1993) Biodistribution of a boronated porphyrin in BC-1 mammary carcinoma. In *Advances in Neutron Capture Therapy*, Soloway AH, Barth RF and Carpenter DE (eds), pp. 505–508. Plenum Publishing: New York

Mishima Y, Honda C, Ichihashi M, Obara H, Hiratsuka J, Karashima H, Kobayashi T, Kanda K and Yoshino K (1989*a*) Treatment of malignant melanoma by single thermal neutron capture therapy with melanoma-seeking $^{10}B$-compound. *Lancet* **2**: 388–389

Mishima Y, Ichihashi M, Hatta S, Honda C; Sasase A, Yamamura K, Kanda K, Kobayashi T and Fukuda H (1989*b*) Selected thermal neutron capture therapy and diagnosis of malignant melanoma – from basic studies to first clinical treatment. In *Clinical Aspects of Neutron Capture Therapy*, Fairchild RG, Bond VP and Woodhead AD (eds), pp. 251–260. Plenum Publishing: New York

Singh M, Ghose T, Faulkner G, Kralovec J and Mezei M (1989) Targeting of methotrexate-conjugating lipsomes with monoclonal antibody against human renal cancer. *Cancer Res* **49**: 3976–3984

Tomita T, Watanabe M, Takahashi T, Kumai K, Tadakuma T and Yasuda T (1989) Temperature-sensitive release of adriamycin, an amphiphilic antitumor agent, from dipalmitoyl-phosphatidyl-choline-cholesterol liposomes. *Biochim Biophys Acta* **978**: 185–190

Yanagië H, Tomita T, Kobayashi H, Fujii Y, Takahashi T, Hasumi K, Nariuchi H and Sekiguchi M (1991) Application of boronated anti-CEA immunoliposomes to tumour cell growth inhibition in *in vitro* boron neutron capture therapy model. *Br J Cancer* **63**: 522–526

© Cancer Research Campaign 1997